# Efficacy and Safety of Atezolizumab and Bevacizumab in Appendiceal Adenocarcinoma

**DOI:** 10.1158/2767-9764.CRC-24-0019

**Published:** 2024-05-29

**Authors:** Nicholas J. Hornstein, Mohammad A. Zeineddine, Betul B. Gunes, Andrew J. Pellatt, Mark Knafl, Haifeng Zhu, Anneleis F. Willett, Abdelrahman Yousef, Suyu Liu, Ryan Sun, Andrew Futreal, Scott E. Woodman, Melissa W. Taggart, Michael J. Overman, Daniel M. Halperin, Kanwal P. Raghav, John Paul Shen

**Affiliations:** 1 Medical Oncology Fellowship, University of Texas MD Anderson Cancer Center, Houston, Texas.; 2 GI Medical Oncology, University of Texas MD Anderson Cancer Center, Houston, Texas.; 3 Faculty of Medicine, Biruni University, Zeytinburnu, Istanbul, Turkey.; 4 Biostatistics, University of Texas MD Anderson Cancer Center, Houston, Texas.; 5 Genomic Medicine, University of Texas M. D. Anderson Cancer Center, Houston, Texas.; 6 Pathology, University of Texas MD Anderson Cancer Center, Houston, Texas.

## Abstract

**Purpose:**

Appendiceal adenocarcinoma (AA) remains an orphan disease with limited treatment options for patients unable to undergo surgical resection. Evidence supporting the efficacy of combined VEGF and PD-1 inhibition in other tumor types provided a compelling rationale for investigating this combination in AA, where immune checkpoint inhibitors have not been explored previously.

**Experimental Design:**

We conducted a prospective, single-arm phase II study evaluating efficacy and safety of atezolizumab in conjunction with bevacizumab (Atezo+Bev) in advanced, unresectable AA.

**Results:**

Patients treated with the Atezo+Bev combination had 100% disease control rate (1 partial response, 15 stable disease) with progression-free survival (PFS) of 18.3 months and overall survival not-yet-reached with median duration of follow-up of 40 months. These survival intervals were significantly longer relative to a clinically and molecularly matched synthetic control cohort treated with cytotoxic chemotherapy designed for colorectal cancer (PFS of 4.4 months, *P* = 0.041).

**Conclusions:**

In light of recent data demonstrating a lack of efficacy of 5-fluorouracil–based chemotherapy, Atezo+Bev is a promising treatment option for patients with low-grade unresectable AA; further study is warranted.

**Significance:**

AA remains an orphan disease with limited systemic therapy options for patients who are not candidates for surgical resection. These data suggest activity from combined VEGF and PD-L1 inhibition that warrants further study.

## Introduction

Appendiceal adenocarcinoma (AA) is an understudied and lethal malignancy with a profound lack of effective systemic treatments. Historically, management principles of appendiceal cancer have been derived from colon adenocarcinoma presumably due to its regional proximity (1, 2) although this generalization ignores clear differences between AA and colorectal cancer in terms of natural history, histologic appearance, as well as somatic mutation and transcriptomic profile (3–5). Appendiceal cancer is unique among gastrointestinal tumors in that metastasis is limited almost exclusively to the peritoneum, with both lymphatic and hematogenous spread uncommon. The majority of tumors have mucinous histology but there is marked diversity in histologic subtypes including non-mucinous (also called colonic-type) goblet cell, and signet ring adenocarcinoma (6). In addition, unlike colorectal cancer, histologic grade is an important predictor of survival in AA and is associated with somatic mutation; *GNAS* mutation is enriched in low-grade and *TP53* mutation is enriched in high-grade tumors (3, 4). Very few prospective trials evaluating systemic therapy in appendiceal cancer have ever been performed (7), and one prospective, randomized crossover study of low-grade AA showed no benefit to 5-fluorouracil (5-FU)-based chemotherapy versus observation in terms of disease progression (8). Despite the lack of data supporting the use of colorectal cancer chemotherapy in AA, the most commonly used drugs in AA are 5-FU, oxaliplatin, irinotecan, and bevacizumab. In the United States, this treatment paradigm is in large part due to guidelines from the National Comprehensive Cancer Network (NCCN) that endorse treating appendiceal cancer similar to colorectal cancer (1), and insurance restrictions preventing the use of non-colorectal cancer drugs. As such, there is a considerable unmet need to develop effective systemic treatments for patients with AA who are unable to undergo cytoreductive surgery. Given the success of the combination of anti-PD-L1 and anti-VEGF antibodies in other malignancies, including hepatocellular carcinoma and peritoneal mesothelioma (9, 10), we hypothesized that this regimen could similarly be effective in AA. Accordingly, we designed a single-arm phase IIA prospective clinical trial to assess the safety and efficacy of atezolizumab in conjunction with bevacizumab (Atezo+Bev) in advanced AA.

## Materials and Methods

This study was designed as an open-label single-arm phase IIA clinical trial; the only site was the University of Texas MD Anderson Cancer Center. Patients were treated with atezolizumab (840 mg i.v.) and bevacizumab (15 mg/kq i.v.) on a 21-day cycle. The primary endpoint was overall response rate (ORR) per RECIST version 1.1 determined by independent radiology review, CT scans were performed every 9 weeks. Key prespecified secondary endpoints included safety, disease control rate (DCR), progression-free (PFS), and overall (OS) survival. Outcomes were assessed by ORR and confidence interval (CI) utilizing the Clopper and Pearson method. Primary inclusion criteria was metastatic AA with extensive tumor burden such that patient was not deemed to be a candidates for cytoreductive surgery as determined by the MD Anderson Peritoneal Surface Malignancy Tumor Board. Additional inclusion criteria included Eastern Cooperative Oncology Group (ECOG) performance status of 0–2, age ≥18 years, measurable disease by RECIST version 1.1 (RECISTv1.1), and appropriate organ function (hepatic, renal, and hematologic). Notable exclusion criteria included clinically symptomatic malignant bowel obstruction, uncontrolled secondary illnesses, additional concomitant malignancy, or history of autoimmune disease. All patients provided written informed consent. This study was conducted in accordance with recognized ethical guidelines including the Declaration of Helsinki and U.S Common Rule, and the study was approved by an Institutional Review Board (IRB).

This study in appendiceal cancer was part of a larger multitumor type collection of trials where in each case cohort size was calculated on the basis of the estimated overall best response rate compared with historical control in the current literature. Unfortunately there were not any prior prospective trials with a published ORR in appendiceal cancer. An ORR of 10% was picked as a conservative estimate of the spontaneous, without treatment response rate. We performed an independent binomial test against the null hypothesis. With a one-sided type I error rate of 0.05, a sample size of 16 patients provided 75% power to detect an ORR of 30% on study compared with a control ORR of 10%.

AA is known for marked interpatient heterogeneity with dramatically different survival times based on histologic grade (3). We therefore sought to determine the historic PFS and OS of standard-of-care (SOC) treatment by leveraging our extensive database of previously treated AA (*n* = 3,715) to generate a synthetic cohort of matched patients. The synthetic cohort was selected on the basis of clinical-pathologic variables previously shown to be associated with outcomes: age, mutation status (*KRAS, GNAS, TP53*), gender, and tumor grade (11). Matching with nearest neighbors was performed with the software package MatchIt; if multiple individuals were equally suitable for inclusion in the synthetic cohort they were randomly chosen (12). After matching each study patient, the line of therapy for the experimental Atezo-Bev in the trial patient was matched to the chemotherapy treatment from same line of therapy in the paired patient. Both PFS and OS for atezolizumab + bevacizumab were determined from the date of start of atezolizumab + bevacizumab. For the synthetic control cohort, PFS is calculated from the start of matched line of treatment until progression.

### Data Availability Statement

Data not already included in Supplementary Tables are available upon request of corresponding authors, pursuant to restrictions of IRB protocol.

## Results

Between April 2017 and July 2020, 16 patients with metastatic AA not amenable to curative surgical resection were enrolled in the study; all patients were included for primary endpoint analysis (Supplementary Fig. S1). Patients enrolled had received between 0 and 8 prior lines of treatment, had ECOG performance status of 0 or 1, and came from a mixture of racial/ethnic groups including Caucasian, Asian, and African-American (Table 1). Prior chemotherapy regimens were most commonly 5-FU, FOLFOX/CapeOX or FOLFIRI, and bevacizumab was half of the time (Supplementary Table S1). Two patients had prior cytoreductive surgery (CRS) and hyperthermic intra-peritoneal chemotherapy (HIPEC) surgery. Regarding histology, which is known to strongly influence outcome in AA, all tumors were of mucinous adenocarcinoma subtype and were either well differentiated (grade I, 56%) or moderately differentiated (grade II, 36%) with only one poorly differentiated tumor (grade III, 6%) using the PSOGI consensus classification system (6). Microscopic evaluation of biopsy-derived tumor tissue demonstrated largely acellular, mucinous tumors consistent with diagnosis of mucinous AA. Notably, lymphocytic infiltration was observed in some specimens (Supplementary Fig. S2). All patients were microsatellite stable, and harbored a mixture of *TP53* (6%), *KRAS* (56%), and *GNAS* (37%) mutations. The synthetic cohort was matched with nearly identical clinical and molecular characteristics.

**TABLE 1:** Patient demographics and molecular status

Trial Cohort						Synthetic Cohort					
	*n*	%		*n*	%		*n*	%		*n*	%
Gender			MSS status			Gender			MSS status		
Female	10	62	MSS	16	100%	Female	11	68	MSS	16	100%
Male	6	38	MSI-high	0	0%	Male	5	32	MSI-high	0	0%
Median age	54 (40–81)		KRAS status			Median age	63 (41–80)		KRAS status		
Tumor grade			KRAS mutated	9	56%	Tumor grade			KRAS mutated	12	75%
I	9	56%	KRAS WT	4	25%	I	10	63%	KRAS WT	4	25%
II	6	38%	KRAS UNK	3	19%	II	5	31%	KRAS UNK	0	0%
III	1	6%	TP53 status			III	1	6%	TP53 status		
Prior lines of treatment	2 (0–8)		TP53 mutated	1	6%	Prior lines of treatment (MLOT)	2 (0–8)		TP53 mutated	0	0%
None	6	38%	TP53 WT	12	75%	None	6	40%	TP53 WT	16	100%
1 Line	2	12%	TP53 UNK	3	19%	1 Line	2	13%	TP53 UNK	0	0%
>1 Line	8	50%	GNAS status			>1 Line	7	47%	GNAS status		
ECOG			GNAS mutated	6	37%	ECOG (MLOT)			GNAS mutated	9	56%
Zero	6	38%	GNAS WT	7	44%	Zero	6	38%	GNAS WT	7	44%
One	10	62%	GNAS UNK	3	19%	One	10	62%	GNAS UNK	0	0%
Ethnicity						Ethnicity					
White	12	75%				White	13	81%			
Asian	1	6%				Asian	2	13%			
Hispanic or Latino	0	0%				Hispanic or Latino	0	0%			
Black or AA	2	12%				Black or AA	1	6%			
Other	1	6%				Other	0	0%			

**FIGURE 1:**
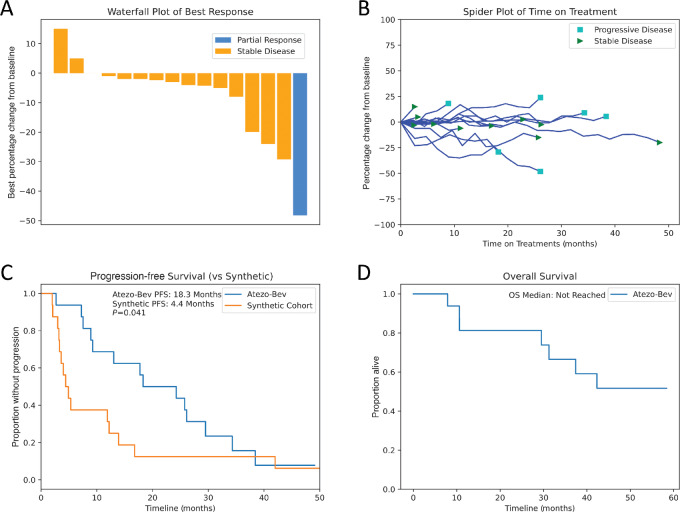
Patient outcomes. **A,** Waterfall plot of best response. **B,** Spider plot demonstrating change from baseline over time on treatment and end of therapy response. **C,** Comparison of combination of Atezo-Bev to matched line of therapy in a synthetic cohort, PFS was 18.3 months (95% CI: 8.9–29.5) versus 4.4 months (95% CI: 3.1–12.2). **D,** OS plot for combination of Atezo-Bev, median not-yet-reached (lower 95% CI > 29.5 months).

All patients received at least one dose of experimental therapy and had posttreatment imaging performed to assess response. The confirmed ORR per RECISTv1.1 was 6.25% (1/16; 1 partial response) with DCR of 100% with 15 patients obtaining stable disease in addition to 1 patient with partial response (Fig. 1A and B). Of note, RECISTv1.1 is known to underestimate response in peritoneal tumors given its reliance on cross-sectional area; however, this study was designed before the development of modifies RECIST for peritoneal disease (8). Median PFS (mPFS) was 18.3 months (95% CI: 8.9–29.5) with median OS not-yet-reached, lower bound of 95% CI > 29.5 months, with a median of 40 months of follow-up. mPFS for the matched line of therapy from synthetic control cohort was only 4.4 months (95% CI: 3.1–12.2), significantly shorter compared with Atezo+Bev (HR = 1.9, *P* = 0.041; Fig. 1C). The mPFS for the control cohort was similar to prior reports for well and moderately differentiated AA (13). Median OS was not-yet-reached, 9 of 16 patients remain alive with median follow-up 60 months (Fig. 1D).

**FIGURE 2:**
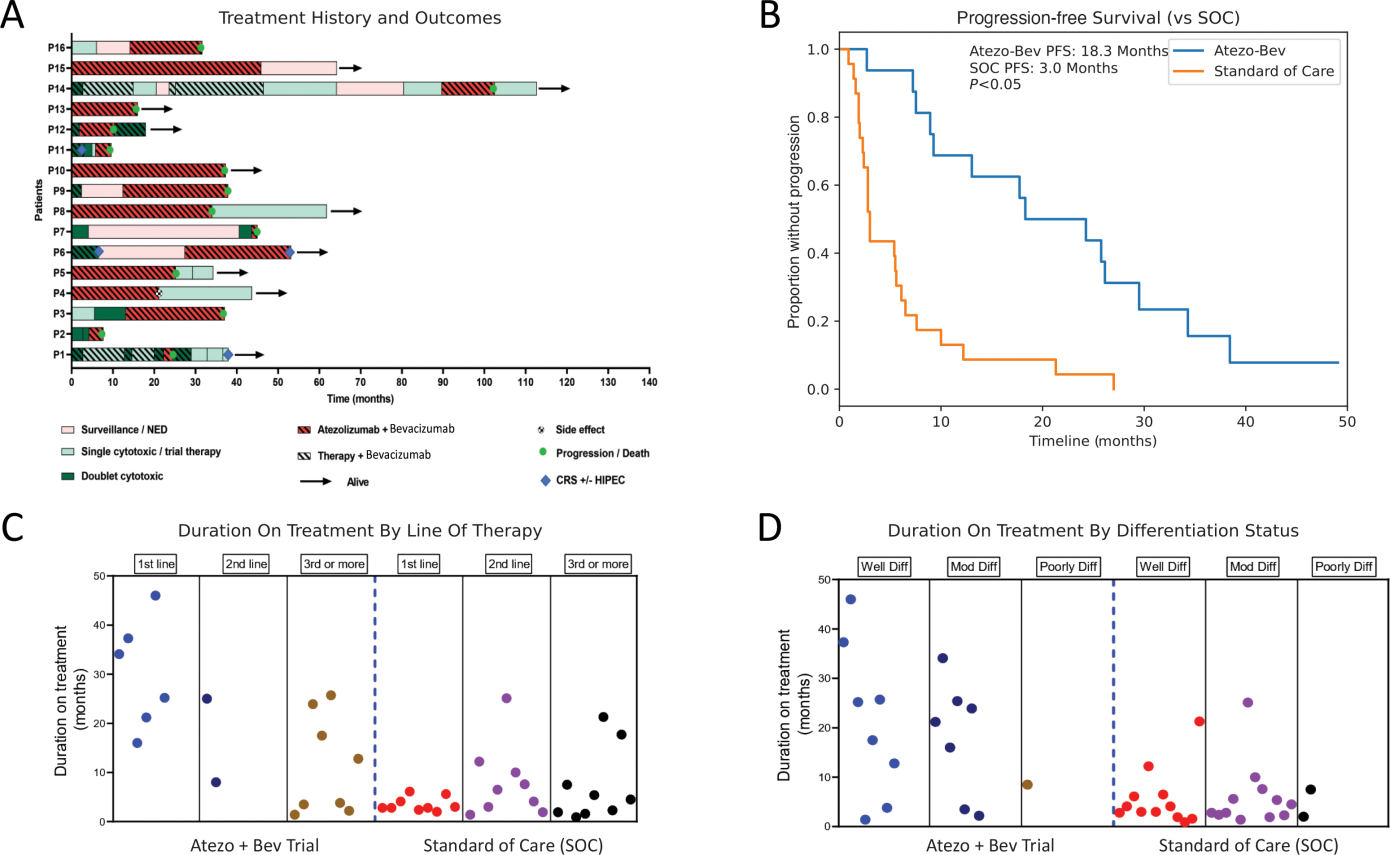
Comparison to synthetic cohort and stratification. **A,** Swimmers plot demonstrating time on treatment for all therapies patient received. **B,** PFS plot demonstrating combination of Atezo-Bev versus median prior SOC treatments 18.3 months (95% CI: 8.9–29.5) versus 3 months (95% CI: 3.1–8.7). **C,** Duration of treatment stratified by line of therapy comparing Atezo-Bev with duration on SOC treatments patients received prior to study enrollment. **D,** Duration of treatment stratified by differentiation status comparing Atezo-Bev with duration on SOC treatments patients received prior to study enrollment.

To further evaluate the relative benefit of Atezo+Bev in this cohort we compared the PFS on study with that of each patient's prior SOC treatments (Fig. 2A). The PFS on Atezo+Bev was significantly longer than SOC, 18.3 versus 3 months (95% CI: 3.1–8.7, *P* < 0.005; Fig. 2B). Importantly, the longer PFS seen with Atezo+Bev compared with prior lines of SOC was consistent irrespective of tumor grade (Fig. 2C). There was a trend toward longer PFS for patients receiving Atezo+Bev in first line, with all 6 of these patients remaining on therapy for greater than a year before progressing. Interestingly, response to first line SOC treatment (either FOLFOX of 5-FU) was particularly short, <6 months, consistent with prior report that 5-FU–based chemotherapy is ineffective in low-grade mucinous appendiceal cancer (8). The PFS for the 8 patients receiving Atezo+Bev as 3rd or greater line of therapy was comparable to that of SOC in similar line of therapy. Duration of Atezo+Bev treatment was similar for patients with well and moderately differentiated tumors (Fig. 2D). Grade 3 treatment-emergent adverse events (no grade 4/5) occurred in 6 (37.5%) patients; most common being syncope (12.5%; Supplementary Table S2). No patients required treatment discontinuation due to side effects.

## Discussion

Because of a lack of prospective clinical trials, AA has historically been treated with 5-FU–based polychemotherapy derived from an abundance of colorectal cancer data. However, recent literature has highlighted the molecular, histopathologic, and clinical differences between AA and colorectal cancer, in addition to the lack of efficacy of these colorectal cancer regimens in well and moderately differentiated AA (5, 8). As such, there is a tremendous unmet need for systemic treatment for patients with advanced, unresectable AA. Bevacizumab has previously been shown to be active when combined with chemotherapy in highly mucinous tumors and is known to be active in peritoneal metastases; notably it has single-agent activity against platinum-resistant ovarian cancer, another tumor with tropism for the peritoneal cavity (14). There is also preclinical evidence supporting the potential role of T cell–stimulating immunotherapies in AA (15, 16). Furthermore, there is growing literature supporting synergist activity from the combination of anti-VEGF and anti-PD-L1 therapies (17). Mechanistically, it has been proposed that VEGF inhibition can upregulate antigen presentation from dendritic cells, decrease proliferation of regulatory T cell, myeloid-derived suppressor cell, and M2-like tumor-associated macrophages, and improve T-cell trafficking and infiltration, all of which contribute to a more favorable tumor microenvironment (18).

In this study, we explored the combination of Atezo+Bev in advanced, unresectable AA. The primary endpoint of ORR by RECISTv1.1 was not met; however, in the years since the trial was designed there has been increasing recognition that standard RECISTv1.1 are inaccurate when applied to measurement of peritoneal disease (8); in retrospect RECIST response was not a good choice of endpoint. The focus should rather be on PFS and OS endpoints, in which the 18.3 months mPFS seen with the experimental Atezo+Bev was more than four times as long as the synthetic control cohort (Fig. 1). With median OS not-yet-reached, it is too early to know whether the PFS benefit will translate into an OS benefit; updated results will be published when available. However, the nearly 14-month extension in PFS in conjunction with a favorable safety profile argues strongly that Atezo+Bev be investigated further in this orphan disease.

Atezo+Bev treatment appeared equally efficacious in both well- and moderately-differentiated tumors; only 1 patient with a poorly-differentiated tumor was enrolled on trial preventing definitive analysis. As expected, the PFS for Atezo+Bev when used in first line was longer then when used as 3rd or greater line. However, the fact that in 3rd or greater line setting Atezo+Bev performed similarly to SOC highlights that Atezo+Bev, especially considering its favorable toxicity profile, should be considered as an additional treatment option in the relapse/refractory setting when the only other alternative is often hospice.

Several inherent limitations to the study design should be noted. Due in part to the rarity of AA, the study was developed with a single arm and without a control cohort, with only 16 total patients. In addition, there was not preplanned stratification based on grade and number of prior therapies; because both factors are known to influence response to treatment the heterogenous nature of the cohort does limit strength of conclusions from these data. An attempt was made to mitigate this limitation by controlling for these factors in the synthetic control cohort. In particular, it is important that these data should not necessarily be extrapolated to patients with poorly-differentiated appendiceal adenocarcinoma, where retrospective data have suggested benefit from cytotoxic chemotherapy.

In conclusion, the combination of Atezo+Bev was well tolerated and demonstrated activity in AA with significant improvement in mPFS relative to control. The combination of PD-L1 and VEGF inhibition should be further studied in AA.

## Supplementary Material

Supplementary Tables Supplementary Table 1 Supplementary Table 2

Supplemental Figure 1 Supplementary Figure 1: Consort Diagram Trial consort diagram

Supplemental Figure 2 Supplementary Figure 2: Representative Pathology Images A,B: Representative images from patient tumor biopsies large mucin deposits and minimal cellularity. Infiltrating lymphocytes are notable in both images.
