# Supplementary material for: Efficacy and Safety of Atezolizumab and Bevacizumab in Appendiceal Adenocarcinoma
Source: Cancer Res Commun. 2024 May 29;4(5):1363–8. doi: 10.1158/2767-9764.CRC-24-0019 (PMC11135244; doi:10.1158/2767-9764.CRC-24-0019)
Supplement: Supplementary Tables — Supplementary Table 1 Supplementary Table 2 [file crc-24-0019-s01.docx]

**Supplemental Table 1: Prior Chemotherapy**

| **Chemotherapy class** | **total # treatments** | **# combined with bevacizumab** |
| --- | --- | --- |
| investigational | 4 | 0 |
| 5FU / Capcitabine | 7 | 3 |
| FOLFOX/CapeOx | 13 | 8 |
| FOLFIRI | 4 | 2 |
| Other single agents | 2 | 2 |

Total numbers of chemotherapy treatments given prior to investigational Atezo-Bev regimen, and number of those treatments that were combined with bevacizumab.

**
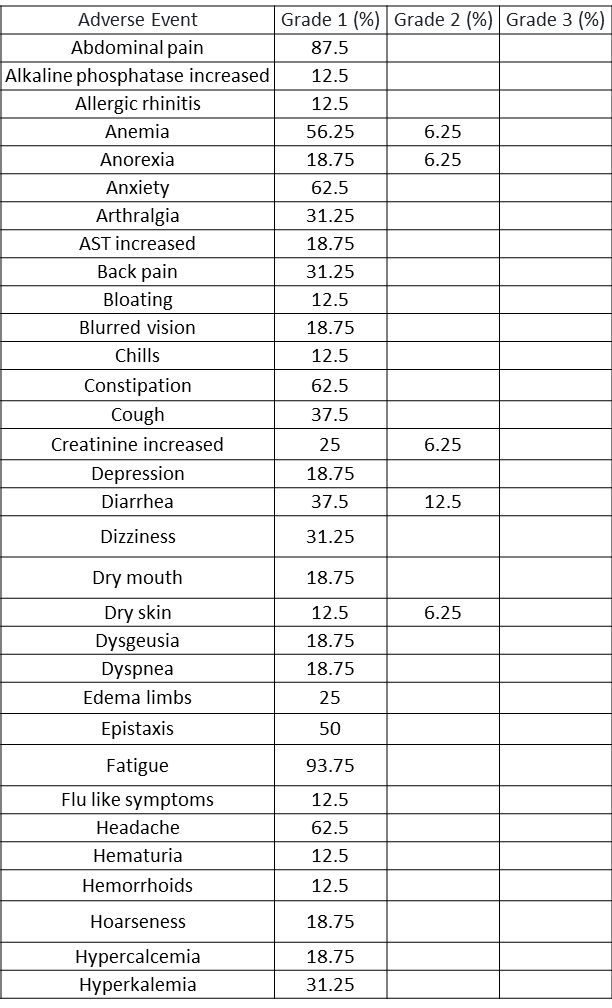

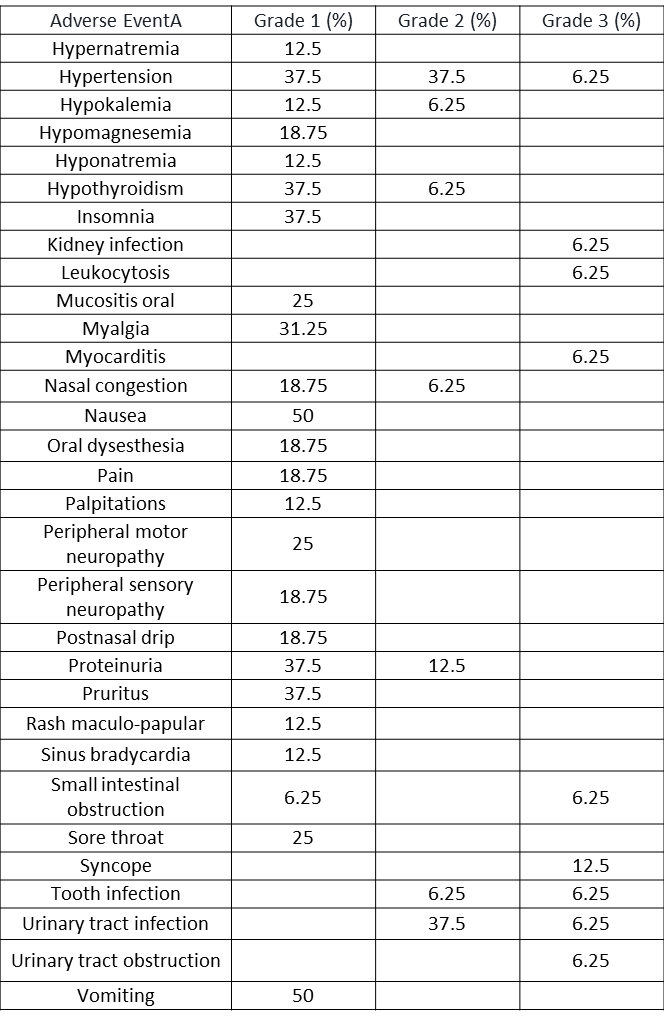
Supplementary Table 2: Adverse Events**

Adverse events were assessed through the duration of the study. The majority of adverse events were grade 1 and self-limited. There were no grade 4 or 5 adverse events.
