# Supplementary material for: Efficacy and Safety of Atezolizumab and Bevacizumab in Appendiceal Adenocarcinoma
Source: Cancer Res Commun. 2024 May 29;4(5):1363–8. doi: 10.1158/2767-9764.CRC-24-0019 (PMC11135244; doi:10.1158/2767-9764.CRC-24-0019)
Supplement: Supplemental Figure 1 — Supplementary Figure 1: Consort Diagram Trial consort diagram [file crc-24-0019-s02.pptx]

## Slide 1
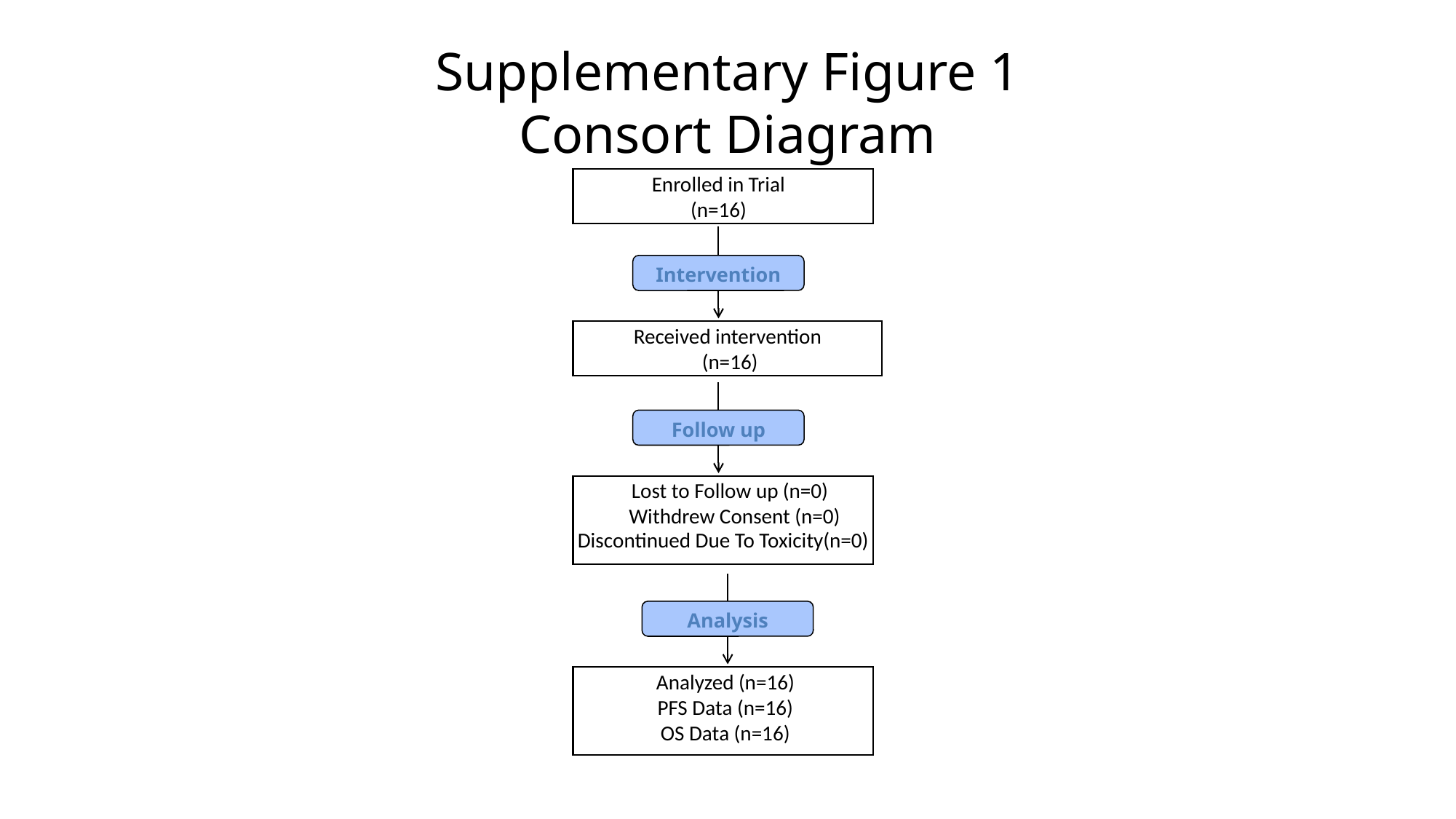

Supplementary Figure 1
Consort Diagram
Enrolled in Trial (n=16)
Intervention
Received intervention
(n=16)
Follow up
Lost to Follow up (n=0)
Withdrew Consent (n=0)
Discontinued Due To Toxicity(n=0)
Analysis
Analyzed (n=16)
PFS Data (n=16)
OS Data (n=16)
