# Supplementary figures and images for: Efficacy and Safety of Atezolizumab and Bevacizumab in Appendiceal Adenocarcinoma
Source: Cancer Res Commun. 2024 May 29;4(5):1363–8. doi: 10.1158/2767-9764.CRC-24-0019 (PMC11135244; doi:10.1158/2767-9764.CRC-24-0019)

## Slide 1
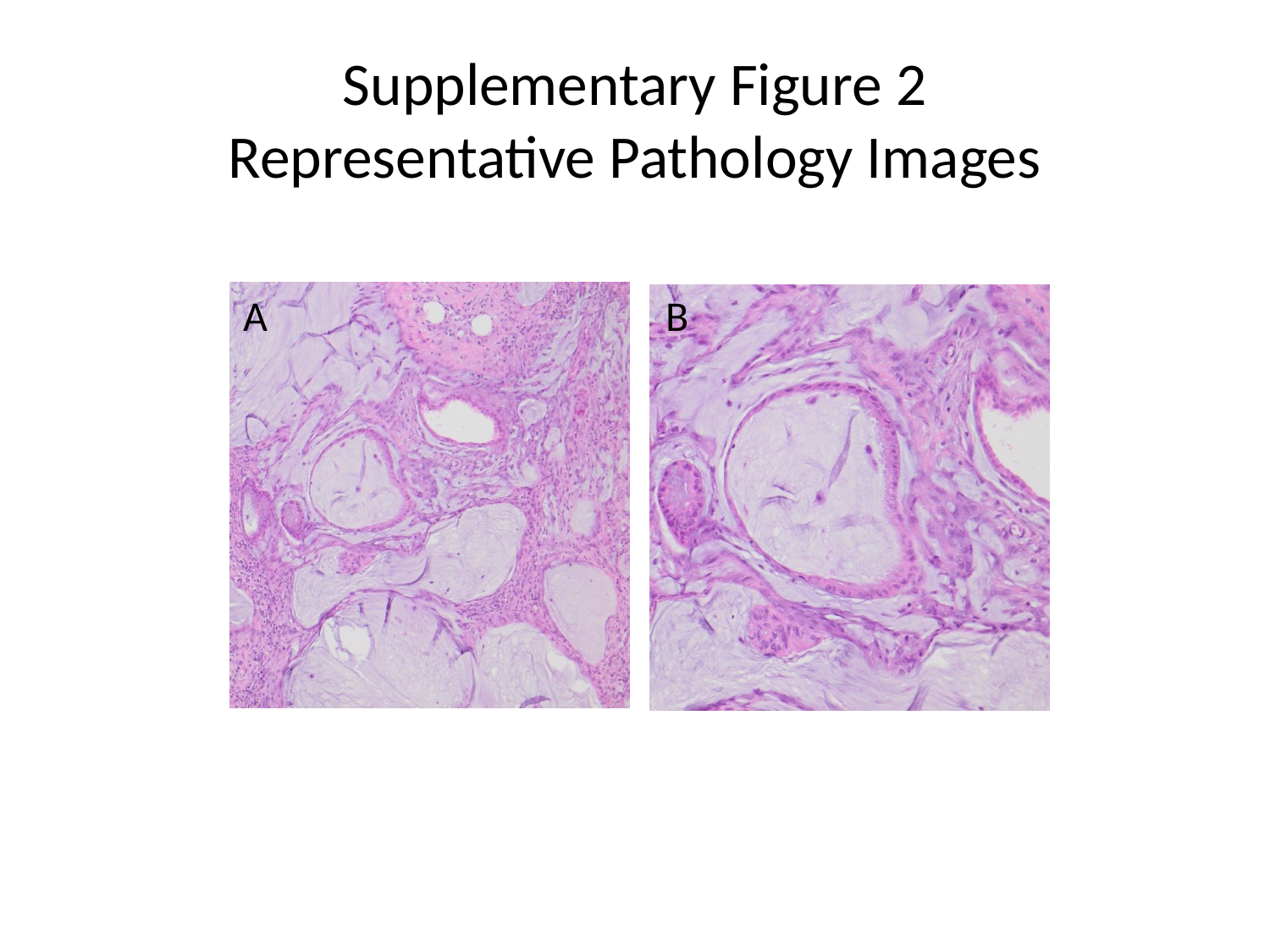

# Supplementary Figure 2 Representative Pathology Images
A
B

Supplement: Supplemental Figure 2 — Supplementary Figure 2: Representative Pathology Images A,B: Representative images from patient tumor biopsies large mucin deposits and minimal cellularity. Infiltrating lymphocytes are notable in both images. [file crc-24-0019-s03.pptx]
